# Prediction models for high risk of suicide in Korean adolescents using machine learning techniques

**DOI:** 10.1371/journal.pone.0217639

**Published:** 2019-06-06

**Authors:** Jun Su Jung, Sung Jin Park, Eun Young Kim, Kyoung-Sae Na, Young Jae Kim, Kwang Gi Kim

**Affiliations:** 1 School of Medicine, Gachon University College of Medicine, Incheon, South Korea; 2 Department of Biomedical Engineering, Gachon University College of Medicine, Incheon, South Korea; 3 Department of Information and Statistics, Korea National Open University, Seoul, South Korea; 4 Department of Radiology, Gil Medical Center, Gachon University College of Medicine, Incheon, South Korea; 5 Department of Psychiatry, Gil Medical Center, Gachon University College of Medicine, Incheon, South Korea; University of Toronto, CANADA

## Abstract

**Objective:**

Suicide in adolescents is a major problem worldwide and previous history of suicide ideation and attempt represents the strongest predictors of future suicidal behavior. The aim of this study was to develop prediction model to identify Korean adolescents of high risk suicide (= who have history of suicide ideation/attempt in previous year) using machine learning techniques.

**Methods:**

A nationally representative dataset of Korea Youth Risk Behavior Web-based Survey (KYRBWS) was used (n = 59,984 of middle and high school students in 2017). The classification process was performed using machine learning techniques such as logistic regression (LR), random forest (RF), support vector machine (SVM), artificial neural network (ANN), and extreme gradient boosting (XGB).

**Results:**

A total of 7,443 adolescents (12.4%) had a previous history of suicidal ideation/attempt. In the multivariable analysis, sadness (odds ratio [OR], 6.41; 95% confidence interval [95% CI], 6.08–6.87), violence (OR, 2.32; 95% CI, 2.01–2.67), substance use (OR, 1.93; 95% CI, 1.52–2.45), and stress (OR, 1.63; 95% CI, 1.40–1.86) were associated factors. Taking into account 26 variables as predictors, the accuracy of models of machine learning techniques to predict the high-risk suicidal was comparable with that of LR; the accuracy was best in XGB (79.0%), followed by SVM (78.7%), LR (77.9%), RF (77.8%), and ANN (77.5%).

**Conclusions:**

The machine leaning techniques showed comparable performance with LR to classify adolescents who have previous history of suicidal ideation/attempt. This model will hopefully serve as a foundation for decreasing future suicides as it enables early identification of adolescents at risk of suicide and modification of risk factors.

## Introduction

In South Korea, suicide in adolescents has been emerging as a major public health problem. The suicide rate has increased annually in adolescents and is recorded as not only one of the highest, but also the most rapidly increasing feature among Organization for Economic Cooperation and Development (OECD) countries.

Although several studies have identified risk factors of suicide [1–5], a recent meta-analysis reveals that the ability to predict suicide behaviors have remained limited [6]. New application of machine learning techniques are gaining attention to identify suicide risk at various clinical setting [7]; Passos et al. classified individuals with a history of suicide attempt among patients with mood disorders based on demographic and clinical data [8]. Oh et al. distinguished suicide attempters from non-suicide attempters among patients with depression or anxiety disorders, applying ANN to multiple psychiatric scales and sociodemographic data [5]. Using general characteristics and insurance data from the National Health Insurance Service cohort in Korea, one recent study analyzed the probability of death by suicide [9].

Since the presence of previous suicide ideation/attempt represent one of the strongest predictors of future suicide behavior and death by suicide [6], it is important to identify adolescents who have history of previous suicide ideation/attempt. Herein, the purpose of this study was to establish prediction models for high-risk of suicide in Korean adolescents using machine learning techniques.

## Materials and methods

### Data collection and preparation

Data used in this study was brought from the Korean Young Risk Behavior Web-based Survey (KYRBWS) XIII in 2017. The KYRBWS is a self-administered online survey and it was approved by the Institutional Review Board (Certificate Number: 11758) of the Korea Centers for Disease Control and Prevention (KCDC).

This survey intends to grasp South Korean adolescents’ health-risk behaviors such as smoking, alcohol use, obesity, physical activity, eating habits, injury prevention, mental health, sexual behaviors, oral health, allergic disorders, personal hygiene, internet addiction, and health equity. Participants were provided with identification numbers and were guaranteed anonymity, and all participants completed an online, self-reported questionnaire in a school computer room after the survey had been fully explained. All data used in this study have been fully anonymized before we accessed them. All procedures and terms and conditions of the survey have been complied with were performed in accordance with the Declaration of Helsinki 7th version and informed consent was obtained from all participants. The test–retest reliability of the KYRBWS questionnaire has been reported to be stable [10]. The dataset and questionnaire is provided with guidelines for calculating a health-related index through the KCDC online site (http://www.cdc.go.kr/CDC/eng/main.jsp).

In 2017, the KYRBWS dataset included a total 62,276 adolescents from 799 middle and high schools (response rate: 95.8%), using a complex sampling design which involves stratification, clustering, and multistage sampling.

### Suicide

High risk of suicide, as a dependent variable, was categorized as adolescents who had either suicidal ideation or suicidal attempt in previous year. Suicidal ideation was defined as a yes response to the question, “Did you consider suicide in the last 12 months?” and suicidal attempt was defined as a yes response to the question, “Did you attempt suicide in the last 12 months?” The respondents who experienced either suicidal ideation or suicidal attempt were categorized within the high risk of suicide group.

### Independent variables

Independent variables included socio-demographic variables (sex, grade, city type, academic achievement, family structure, family socioeconomic status, and education level of father and mother), health-related lifestyle factors (current smoking, current alcohol consumption, substance use, physical activity, obesity, sexual experience, and internet addiction), and psychological stress factors (sadness, stress, self-rated health, sleep satisfaction, self-rated weight, distorted weight perception, school injury, and violence). Comorbidities included asthma, allergic rhinitis, and atopic dermatitis.

School grade was divided as middle school (Grades 1–3, corresponding age 12–15 years) and high school (Grades 4–6, corresponding age 16–18 years). City type was categorized as big cities, small and medium-sized cities, and countryside. Academic achievement was categorized as high, high middle, middle, low middle, and low. Family structure was categorized as having both parents, having either parent, and neither parent. Family socioeconomic status (SES) was categorized as high, high middle, middle, low middle, and low. Education level of father and mother was categorized as unknown, middle school graduate or less, high school graduate, and college or graduate degree.

Current smoking, current alcohol consumption, and substance use were defined as a yes response to the questions: “Did you smoke or drink alcohol more than once within the last 30 days?” and “Have you ever used any substance or sniffed glue or butane habitually on purpose?”

Physical activity was categorized as “active” (vigorous physical activities more than two days among the last seven days) or “inactive.” Vigorous physical activities were defined as those that make one sweat or feel breathless for 20 minutes or more in the questionnaire.

Body mass index (BMI) was calculated based on the self-reported height and weight, and was categorized as underweight (≤ 5^th^ percentile), normal (5-85^th^ percentile), overweight (85-95^th^ percentile), and obesity (≥ 95^th^ percentile or BMI ≥ 25 kg/m^2^). Self-rated weight was categorized as very fat, fat, normal, thin, and very thin. Distorted weight perception was defined when respondents answered “very fat” or “fat” for the self-rated weight question, while his or her actual weight was categorized as underweight or normal.

Information regarding sexual experience, school injury, and internet addiction was also collected. For sadness, the adolescents were asked, “In the last 12 months, has a feeling of sadness interrupted your daily activities for at least two weeks?” In addition, stress, self-rated health, and sleep satisfaction were categorized in five levels by the extent of these symptoms.

### Models to predict high risk of suicide

**Fig 1:**
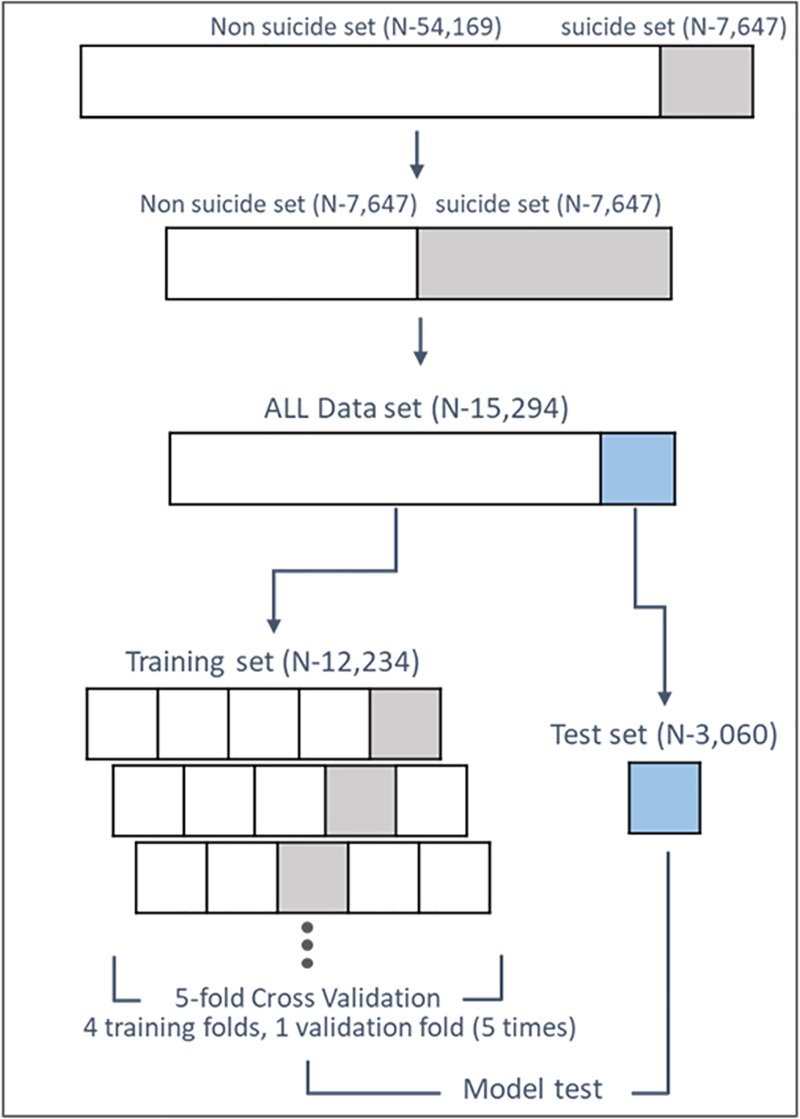
Scheme prediction model development.

To prevent learning bias resulting from an imbalanced dataset (the proportion of the non-suicide group was about 7 times larger than the suicide group in the entire dataset), a balanced dataset (same number of age- and sex-matched non-suicide group for the suicide group, n = 7,647 for each group) was selected from preprocessed data in terms of down-sampling (Fig 1). To prevent overfitting, the preprocessed dataset was split in five equally-sized random groups using a 5-fold cross validation. One group was used as the test set and the other groups were used as the training sets for the machine learning prediction models. Five machine learning methods were trained: logistic regression (LR), random forest (RF), support vector machine (SVM), artificial neural network (ANN), and extreme gradient boosting (XGB). Optimal parameters for each machine learning method were selected through a grid search (Table 1). The variables used in the model were categorical; hence, a 0 or 1 value was applied by one-hot encoding.

**Table 1:** Optimal parameters for each machine learning model are selected through the grid search.

Model	Optimal parameters
LR	Penalty: ‘l2,’ C: 0.1
SVM	C: 0.1, gamma: 0.01, kernel: ‘rbf’
RF	n_estimators: 3000, max depth: 5, min samples leaf: 4, min samples split: 10
ANN	Optimizer: ‘Adam’, learning rate: 0.0001, batch size: 200, epoch: 60
XGB	n_estimators: 5000, learning rate: 0.05, colsample bytree: 0.3, max depth: 4, gamma: 1, lambda: 0.5, alpha: 0.5

A comparison of LR and other machine learning discriminations for each model was performed, in terms of sensitivity, specificity, positive predictive value (PPV), negative predictive value (NPV), and accuracy to predict adolescents who had a history of suicidal ideation or attempt. For test dataset, the area under the receiver operating characteristic curve (AUC) for each model was also calculated to evaluate general prediction performance.

### Statistical analysis

Results are presented as percentages for categorical variables and as means (± standard deviation) for continuous variables. Categorical variables and continuous variables were compared using the chi-square test or the Student’s *t*-test for comparisons between adolescents with/without risk of suicide. Multivariate regression analysis was used to identify factors associated with previous suicidal ideation or attempt using the backward stepwise selection method.

The analysis and machine learning models and diagnostic performance was evaluated using the open-source statistical software Python version 3.6.0. *P*-values of less than 0.05 (two-sided) were considered significant.

## Results

The clinical characteristics for a total of 59,984 subjects with valid information regarding previous history of suicidal ideation/attempt are summarized in Table 2. The high risk suicide group showed higher proportions of girl, low school grade, low academic achievement, those not living with both parents, low family SES, low parental education level, current smoking, current alcohol drinking, substance use, inactive physical activity, sexual experience, internet addiction, sadness, high stress, poor self-rated health, low sleep satisfaction, high self-rated weight, distorted weight perception, experience of school injury and violence, and presence of comorbid diseases (asthma, allergic rhinitis, atopic dermatitis).

**Table 2:** Characteristics of high-risk suicide (*n* = 7,443) and no high-risk suicide (*n* = 52,541).

	no high-risk suicide (n *=* 52,541)	high-risk suicide (n = 7,443)	*P* value*
Sex, boy	27493 (52.3%)	2891 (38.8%)	<0.001
Age (yrs.)	15.0±1.7	15.0±1.8	0.695
School			<0.001
Middle school	25876 (49.2%)	3869 (52.0%)	
High school	26665 (50.8%)	3574 (48.0%)	
School grade			<0.001
G1	8800 (16.7%)	1039 (14.0%)	
G2	8520 (16.2%)	1435 (19.3%)	
G3	8556 (16.3%)	1395 (18.7%)	
G4	8760 (16.7%)	1057 (14.2%)	
G5	9071 (17.3%)	1327 (17.8%)	
G6	8834 (16.8%)	1190 (16.0%)	
City type			0.654
countryside	4094 (7.8%)	563 (7.6%)	
small/medium-sized cities	25154 (47.9%)	3545 (47.6%)	
big cities	23293 (44.3%)	3335 (44.8%)	
Academic achievement			<0.001
high	7221 (13.7%)	878 (11.8%)	
high middle	13830 (26.3%)	1632 (21.9%)	
middle	15286 (29.1%)	1926 (25.9%)	
low middle	11439 (21.8%)	1922 (25.8%)	
low	4765 (9.1%)	1085 (14.6%)	
Family structure			<0.001
live with both parents	43647 (83.1%)	5740 (77.1%)	
live with one parent	4955 (9.4%)	938 (12.6%)	
neither parent	3939 (7.5%)	765 (10.3%)	
Family SES			<0.001
high	5663 (10.8%)	700 (9.4%)	
high middle	15518 (29.5%)	1987 (26.7%)	
middle	24500 (46.6%)	3103 (41.7%)	
low middle	5790 (11.0%)	1260 (16.9%)	
low	1070 (2.0%)	393 (5.3%)	
Education, father			<0.001
unknown	11405 (21.7%)	1614 (21.7%)	
middle school graduate or less	932 (1.8%)	190 (2.6%)	
high school graduate	13488 (25.7%)	1902 (25.6%)	
college or graduate degree	26716 (50.8%)	3737 (50.2%)	
Education, mother			<0.001
unknown	10695 (20.4%)	1515 (20.4%)	
middle school graduate or less	779 (1.5%)	175 (2.4%)	
high school graduate	16530 (31.5%)	2254 (30.3%)	
college or graduate degree	24537 (46.7%)	3499 (47.0%)	
Current smoking (yes)	2748 (5.2%)	753 (10.1%)	<0.001
Current alcohol drinking (yes)	7474 (14.2%)	1659 (22.3%)	<0.001
Substance use (yes)	303 (0.6%)	196 (2.6%)	<0.001
Physical activity			<0.001
active	20243 (38.5%)	2689 (36.1%)	
inactive	32298 (61.5%)	4754 (63.9%)	
Body mass index (kg/m^2^)	21.1±3.4	21.2±3.4	0.033
Obesity			0.228
underweight	4088 (7.8%)	621 (8.3%)	
normal	39827 (75.8%)	5648 (75.9%)	
overweight	1326 (2.5%)	178 (2.4%)	
obesity	7300 (13.9%)	996 (13.4%)	
Sexual experience (yes)	2128 (4.1%)	616 (8.3%)	<0.001
Internet addiction (yes)	1766 (3.4%)	563 (7.6%)	<0.001
Sadness (yes)	9548 (18.2%)	5389 (72.4%)	<0.001
Stress			<0.001
very high	3648 (6.9%)	2545 (34.2%)	
high	13050 (24.8%)	3086 (41.5%)	
middle	23913 (45.5%)	1507 (20.2%)	
low	9621 (18.3%)	227 (3.0%)	
very low	2309 (4.4%)	78 (1.0%)	
Self-rated health			<0.001
very good	15525 (29.5%)	1204 (16.2%)	
good	23892 (45.5%)	2695 (36.2%)	
normal	10561 (20.1%)	2336 (31.4%)	
poor	2438 (4.6%)	1081 (14.5%)	
very poor	125 (0.2%)	127 (1.7%)	
Sleep satisfaction			<0.001
very high	4570 (8.7%)	323 (4.3%)	
high	9869 (18.8%)	755 (10.1%)	
middle	17375 (33.1%)	1975 (26.5%)	
low	14392 (27.4%)	2428 (32.6%)	
very low	6335 (12.1%)	1962 (26.4%)	
Self-rated weight			<0.001
very thin	2144 (4.1%)	351 (4.7%)	
thin	11176 (21.3%)	1409 (18.9%)	
normal	19141 (36.4%)	2281 (30.6%)	
fat	16914 (32.2%)	2667 (35.8%)	
very fat	3166 (6.0%)	735 (9.9%)	
Distorted weight perception (yes)	16701 (31.8%)	2867 (38.5%)	<0.001
School injury (yes)	12105 (23.0%)	2382 (32.0%)	<0.001
Violence (yes)	893 (1.7%)	529 (7.1%)	<0.001
Asthma (yes)	4343 (8.3%)	827 (11.1%)	<0.001
Allergic rhinitis (yes)	18073 (34.4%)	2906 (39.0%)	<0.001
Atopic dermatitis (yes)	12839 (24.4%)	2152 (28.9%)	<0.001

Note. Values are means ± standard deviation, median (range), or number (percentages).

*Chi-squared test or Student's t test.

SES: socio-economic status

A multivariate regression analysis was performed to identify factors associated with high risk of suicide (Table 3). Sadness (odds ratio [OR], 6.41; 95% confidence interval [95% CI], 6.08–6.87), violence (OR, 2.32; 95% CI, 2.01–2.67), substance use (OR, 1.93; 95% CI, 1.52–2.45), and stress (OR, 1.63; 95% CI, 1.40–1.86) showed relatively strong associations with previous suicide ideation/attempt. There were other factors that showed associations with suicide: girl sex, grade, academic achievement, family structure, family SES, parental education level, current smoking, current alcohol drinking, physical activity, overweight, self-rated health, sleep satisfaction, sexual experience, school injury, and violence.

**Table 3:** Multivariate logistic regression analysis to identify factors associated with high risk of suicide.

	Adjusted OR	(95% CI)	*P* value
Sex			
boy	Reference		
girl	1.250	1.174 to 1.330	<0.001
School grade			
G1	Reference		
G2	0.911	0.829 to 1.000	0.051
G3	0.767	0.697 to 0.844	<0.001
G4	0.531	0.479 to 0.590	<0.001
G5	0.532	0.480 to 0.589	<0.001
G6	0.447	0.403 to 0.497	<0.001
City type			
countryside	Reference		
small/medium-sized cities	0.655	0.595 to 0.720	<0.001
big cities	0.674	0.612 to 0.741	<0.001
Academic achievement			
high	Reference		
high middle	0.766	0.695 to 0.844	<0.001
middle	0.801	0.728 to 0.882	<0.001
low middle	0.909	0.824 to 1.004	0.059
low	0.859	0.765 to 0.964	0.010
Family structure			
live with both parents	Reference		
live with one parent	1.116	1.020 to 1.222	0.017
neither	1.081	0.971 to 1.204	0.155
Family SES			
high	Reference		
high middle	0.822	0.743 to 0.909	<0.001
middle	0.804	0.728 to 0.882	<0.001
low middle	1.023	0.910 to 1.152	0.699
low	1.094	0.920 to 1.300	0.308
Education, father			
unknown	0.844	0.767 to 0.929	0.001
middle school graduate or less	1.003	0.817 to 1.230	0.981
high school graduate	0.967	0.894 to 1.046	0.406
college or graduate degree	Reference		
Education, mother			
unknown	0.911	0.827 to 1.005	0.062
middle school graduate or less	1.075	0.868 to 1.331	0.508
high school graduate	0.852	0.790 to 0.918	<0.001
college or graduate degree	Reference		
Current smoking (yes)	1.235	1.097 to 1.391	<0.001
Current alcohol drinking (yes)	1.184	1.093 to 1.282	<0.001
Substance use (yes)	1.932	1.523 to 2.450	<0.001
Physical activity			
active	Reference		
inactive	0.879	0.827 to 0.935	<0.001
Obesity			
normal	Reference		
underweight	1.089	0.980 to 1.210	0.113
overweight	0.767	0.636 to 0.924	0.005
obesity	0.937	0.862 to 1.018	0.122
Sexual experience (yes)	1.193	1.054 to 1.351	0.005
Internet addiction (yes)	1.230	0.911 to 1.660	0.177
Sadness (yes)	6.464	6.083 to 6.868	<0.001
Stress			
very high	1.626	1.398 to 1.892	<0.001
high	0.843	0.729 to 0.975	0.021
middle	0.360	0.311 to 0.416	<0.001
low	0.182	0.151 to 0.218	<0.001
very low	Reference		
Self-rated health			
very good	Reference		
good	1.116	1.030 to 1.208	0.007
normal	1.537	1.409 to 1.677	<0.001
poor	2.009	1.794 to 2.249	<0.001
very poor	2.901	2.131 to 3.950	<0.001
Sleep satisfaction			
very high	Reference		
high	0.690	0.604 to 0.788	<0.001
middle	0.777	0.688 to 0.877	<0.001
low	0.851	0.753 to 0.961	0.010
very low	0.883	0.776 to 1.005	0.059
Self-rated weight			
very thin	Reference		
thin	0.446	0.395 to 0.503	<0.001
normal	0.426	0.380 to 0.478	<0.001
fat	0.501	0.421 to 0.598	<0.001
very fat	0.578	0.476 to 0.703	<0.001
Distorted weight perception (yes)	0.967	0.828 to 1.129	0.671
School injury (yes)	1.078	1.012 to 1.148	0.020
Violence (yes)	2.317	2.014 to 2.666	<0.001
Asthma (yes)	1.022	0.929 to 1.124	0.653
Allergic rhinitis (yes)	0.988	0.930 to 1.050	0.702
Atopic dermatitis (yes)	1.053	0.987 to 1.122	0.117

Note. SES: socio-economic status

**Fig 2:**
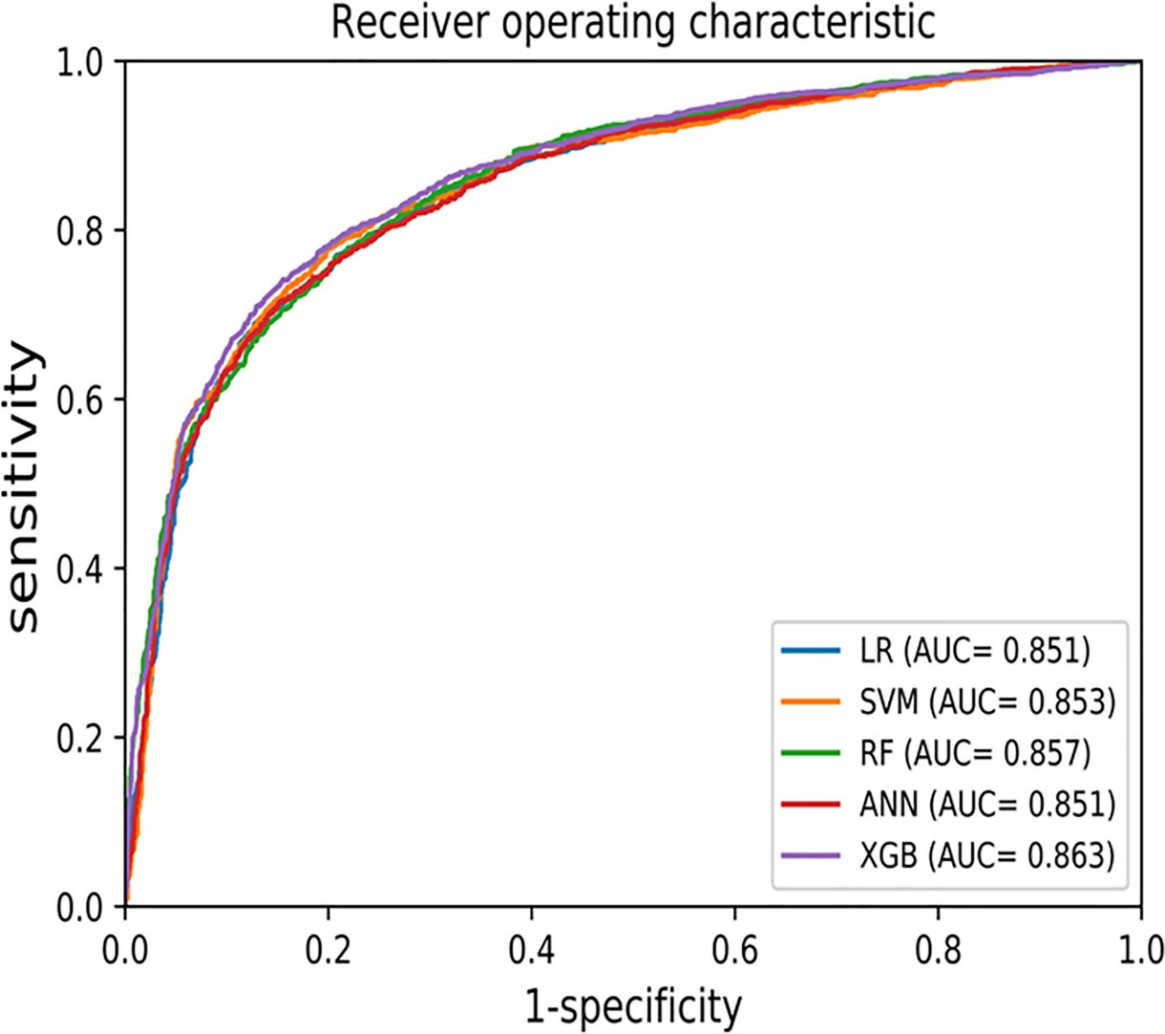
Receiver operating characteristic (ROC) curve.

For the test dataset, the confusion matrix and receiver operating characteristic (ROC) curve show that the diagnostic performance of machine learning techniques are comparable with that of the LR result (Table 4 and Fig 2). XGB showed the best performance, with a sensitivity of 78.5%, specificity of 79.4%, PPV of 79.2%, NPV of 78.7%, classification accuracy of 79.0%, and AUC of 0.863.

**Table 4:** Confusion matrix for prediction models (Test set).

	Model	Sensitivity	Specificity	PPV	NPV	Accuracy	AUC
**Test set**	LR	78.2%	77.6%	77.7%	78.0%	77.9%	0.851
	SVM	78.4%	78.9%	78.8%	78.5%	78.7%	0.853
	RF	77.5%	78.0%	77.9%	77.6%	77.8%	0.857
	ANN	77.3%	77.8%	77.7%	77.4%	77.5%	0.851
	XGB	78.5%	79.4%	79.2%	78.7%	79.0%	0.863

Note. LR: logistic regression; SVM: support vector machine; RF: random forest; ANN: artificial neural network; XGB: extreme gradient boosting; PPV: positive predictive value; NPV: negative predictive value; AUC: area under ROC curve

## Discussion

Machine learning techniques offer promise to improve risk prediction for suicide. A systematic review revealed greater prediction accuracy of self-injurious thoughts and behaviors than in previous studies using traditional statistical methods [7].

Machine learning techniques have advantages beyond traditional statistical approaches in psychological research [11]. For example, traditional approaches greatly minimize the number of variables and impose linearity on relationships that likely have more complex associations. On the other hand, machine learning approaches enable the simultaneous testing of numerous variables and their complex interactions and allow for non-linearity in producing predictive models [11].

The purpose of this study was to develop models to determine adolescent at risk of suicide using nationally representative survey dataset in Korea by using machine learning methods. In this study, we applied the LR method and several other machine learning algorithms, and XGB showed the best performance in the test dataset with an accuracy of 79.0% (AUC = 0.863). XGB, one of the machine learning techniques, is highly efficient and flexible and can be easily used on distributed platforms for further computational efficiency [12]. Ensemble learning is possible by attaching another algorithm to XGB. Future studies would possibly show a better performance if XGB is combined with various algorithms rather than a single algorithm model.

However, the machine leaning techniques showed an overall comparable diagnostic performance with LR. The main reason might be due to the type of dataset used in the present study. The KYRBWS survey data are composed of general health-risk behaviors and we arbitrarily select 26 categorical variables to develop prediction models. Further study is warranted to explore the increasing accuracy using latent variables.

The present study has several limitations. First, the KYRBWS was developed to cover general health-risk behaviors including psychological status and previous suicidal behavior, which were examined by simple questions and scales. If the survey had been composed of more detailed questions regarding suicide behavior or psychological status, the performance of models might have improved. Second, this model was developed using the KYRBWS dataset, it does not guarantee the same diagnostic performance with other datasets or populations. In the present study, we used pairing cross validation for imbalance outcome to avoid the problem of “limited generalization” or “overfitting.” Nevertheless, despite these limitations, this is the first study to adopt machine learning techniques to a nationally representative, and large number (n = 59,984) of Korean adolescents.

In conclusion, this study showed that machine learning techniques have the potential to identify Korean adolescents at risk of suicide using nationally representative survey dataset of general health-risk behaviors. Several machine learning models have comparable performance with the conventional LR method, which have potential for development. Establishment of accurate prediction models through additional studies would facilitate early screening of high risk adolescents and correction of modifiable risk factors, so that society can prevent future suicidal behavior and death by suicide.
